# In Search of a Function for the N6-Methyladenosine in Epitranscriptome, Autophagy and Neurodegenerative Diseases

**DOI:** 10.3390/neurolint15030062

**Published:** 2023-08-10

**Authors:** Naoko Suga, Yuka Ikeda, Sayuri Yoshikawa, Kurumi Taniguchi, Haruka Sawamura, Satoru Matsuda

**Affiliations:** Department of Food Science and Nutrition, Nara Women’s University, Kita-Uoya Nishimachi, Nara 630-8506, Japan

**Keywords:** N6-methyladenine, reactive oxygen species, RNA-binding protein, Alzheimer’s disease, neurodegenerative disease

## Abstract

Changes in epitranscriptome with N6-methyladenine (m6A) modification could be involved in the development of multiple diseases, which might be a prevalent modification of messenger RNAs (mRNAs) in eukaryotes. The m6A modification might be performed through the action of methyltransferases, demethylases, and methylation-binding proteins. Importantly, the m6A methylation may be associated with various neurological disorders including Alzheimer’s disease (AD), Parkinson’s disease (PD), depression, aging-related diseases, and/or aging itself. In addition, the m6A methylation might functionally regulate the eukaryotic transcriptome by influencing the splicing, export, subcellular localization, translation, stability, and decay of mRNAs. Neurodegenerative diseases may possess a wide variety of phenotypes, depending on the neurons that degenerate on occasion. Interestingly, an increasing amount of evidence has indicated that m6A modification could modulate the expression of autophagy-related genes and promote autophagy in neuronal cells. Oxidative stresses such as reactive oxygen species (ROS) could stimulate the m6A RNA methylation, which may also be related to the regulation of autophagy and/or the development of neurodegenerative diseases. Both m6A modification and autophagy could also play critical roles in regulating the health condition of neurons. Therefore, a comprehensive understanding of the m6A and autophagy relationship in human diseases may benefit in developing therapeutic strategies in the future. This paper reviews advances in the understanding of the regulatory mechanisms of m6A modification in the occurrence and development of neurodegenerative diseases and/or aging, discussing the possible therapeutic procedures related to mechanisms of m6A RNA methylation and autophagy.

## 1. Introduction

Neurodegenerative diseases are interlaced with the disorders of multicellular function and/or interaction in the central nervous system (CNS) [1]. “Neurodegeneration” represents a condition where neuronal cells gradually lose their potential functions, and eventually perish. Alzheimer’s disease (AD) is the most common neurodegenerative disease. Neurodegenerative diseases have a huge impact on patients themselves, adding to the social economic burden [2], which is regrettably expected to increase worldwide [3]. Now, progress in resolving the mechanisms behind several neurodegenerative diseases has been considerably recognized. For example, epigenetic modifications and genetic risk factors may be implicated in their appearance, and their mixture could predict or support the identification of individuals at significant risk [4]. In general, neurodegenerative diseases could result in motor dysfunctions and behavioral manifestations such as ataxia, and/or dementia [5]. Clarification of pathogenic mechanisms and new targeted drugs have been urgently needed.

The most important advances in RNA-modification-mediated regulation of gene expression might be an evolving field of epitranscriptomics. The epitranscriptome may contain all the biochemical modifications of the RNA within cells, which is dynamically regulated by specific enzymatic reactions. These RNA modifications could regulate a variety of physiological RNA functions. More than hundred chemical alterations of RNAs have been found in all types of RNAs [6]. The most notable RNA methylation may account for more than 60% of all RNA alterations. Among them, the N6-methyladenosine (m6A) might be a noticeable post-transcriptional RNA modification in eukaryotes, which could regulate the expression of various genes [7,8]. It is noteworthy that the m6A is highly enriched in adult brain tissue [9], which might indicate that it plays a critical role in neurogenesis, neurodevelopment, and/or neurodegenerative disorders [10]. In addition, the profusion of m6A in the brain seems to gradually increase with age from newborns, and reaches a peak in later life [11]. Within the structure of total RNAs, the m6A is mostly dispersed in the 3’ untranslated region (UTR), coding sequence of message RNA (mRNA), and/or regions nearby the stop codon [12]. The roles of the m6A modification in the control of gene expression may be strictly connected to various normal and/or pathological routes containing DNA damage response, cellular differentiation, and/or the occurrence of neurodegenerative diseases. In addition to the regulation of the transcriptional mRNA, m6A modification could also regulate the transcription of a variety of non-coding RNAs (ncRNAs) including microRNAs (miRNAs), circular RNAs (circRNAs), and/or long non-coding RNAs (lncRNAs) [13]. Therefore, m6A modification could take part in the regulation of various processes in physiological cellular activities including cell development, embryonic development and/or stress responses, as well as in a regulatory role of several diseases [14,15]. The motif of m6A during the development may diverge across tissues and/or developmental courses [16], in which the m6A modification may be inclined to the coding sequence and/or 3′UTR [16]. Remarkably, even a single modification could have a vast effect on the function of RNAs. Interestingly, ncRNAs such as lncRNAs could also regulate the expression of m6A-related proteins [17].

**Figure 1:**
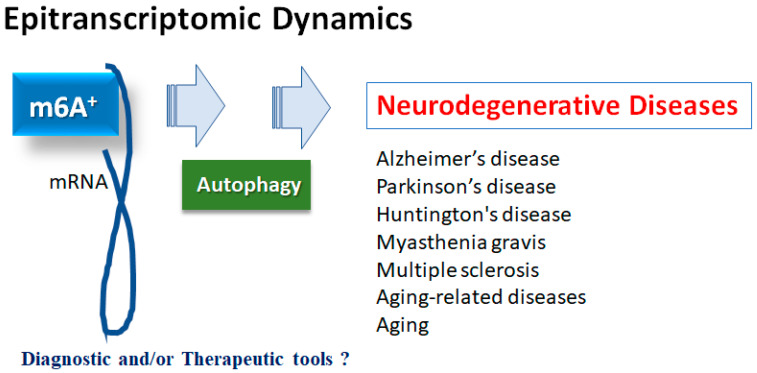
Illustration of the relation of m6A RNAs methylation to various neurodegenerative diseases. Roles of m6A RNAs methylation with autophagy have been proposed in several neurodegenerative diseases including Alzheimer’s disease, Parkinson’s disease, Huntington’s disease, myasthenia gravis and multiple sclerosis, as well as in aging-related diseases and aging. Consequently, m6A RNAs methylation could be diagnostic and/or therapeutic tools for these diseases.

Studies have suggested that m6A modification on mRNAs could influence the proliferation and/or differentiation of neural progenitor cells in a comprehensive understanding of RNA methylation-based diagnosis and/or therapies for neurodegenerative diseases [18,19]. In addition, many studies have also demonstrated the effects of m6A modification in the autophagy mechanisms [20], suggesting that the m6A change might play a critical role in controlling the development of neurodegenerative diseases via the control of autophagy. For example, the m6A modification could instruct direct inhibitory effects on autophagy [21]. Moreover, the m6A modification could influence the construction of autophagosomes to dysregulate autophagy [22]. Occasionally, the m6A modification could even initiate and/or promote autophagy [23]. Interestingly, the properties of the m6A modification for autophagy may be reliant on disease situation and/or stages. Accordingly, abnormal m6A methylation could modify biological processes and/or regulate various human disorders [24]. Therefore, the roles of m6A modifying enzymes might be involved in the pathological process of neurodegenerative diseases, which might be of huge importance to the development of a novel tactic for specific therapy of neurodegenerative diseases. In this report, we will go over the main points of the latest studies describing the impact on m6A modification in neurodegenerative diseases via the regulation of autophagy, and discuss the role of the m6A modification–autophagy relationship for the promising treatment of neurodegenerative diseases (Figure 1). 

## 2. m6A Methylation of mRNAs in Neurodegenerative Diseases

Several studies have shown that m6A methylation could play an important role in the control of the neuron degeneration progression, which might mediate the incidence and/or development of multiple neuron degeneration-related disorders [25]. For example, a number of studies have revealed that m6A is aberrantly expressed in the brain of AD patients [26]. It may be beneficial to explore the pathogenesis of AD, which might contribute to discovering new biomarkers from the perspective of the m6A modification process [27]. As for the process, the m6A is a ubiquitous mRNA modification in eukaryotes, which regularly occurs through the action of methyltransferases, demethylases, and/or methylation-binding proteins. To this point, the m6A methylation of RNAs may be associated with various neurological disorders, including not only AD but also Parkinson’s disease (PD), Huntington’s disease (HD), myasthenia gravis (MG), multiple sclerosis (MS), depression, brain injury, epilepsy, and so forth [28]. Remarkably, it has been suggested that autophagy may be involved in these neurological disorders as well as aging-related diseases [24,28]. Epigenetic modifications including m6A methylation could also play an understandable role in autophagy regulation [29]. For instance, m6A modification has been described to exactly control the expression of autophagy-related genes [30], which might modulate the cellular autophagy levels that may be involved in the development of various diseases including neurodegenerative diseases (Figure 1). Therefore, the m6A modification is known to play a crucial role in various aging-related diseases and/or aging.

### 2.1. Alzheimer’s Disease

The m6A abnormality is directly related to AD [31], which may be one of the leading reasons of dementia and an increasing health problem worldwide. There is still no cure for AD. AD may be characterized by synaptic loss, hyper-phosphorylation of tau, extracellular plaques of amyloid-beta accumulation, and various levels of neuro-inflammation, as in neurodegenerative diseases [32]. Several biological processes may be exaggerated by epitranscriptomic modifications, which could control the expression of specific genes via the metabolism of mRNAs. In response to modifications of certain mRNAs by m6A methylation, the nervous system could become dysfunctional, which might play an important role in the development of AD. In general, the N6-methyladenosine levels are high in the brain [33]. Correspondingly, the expression of multiple m6A methylation regulators could change when AD occurs. In particular, m6A methylation levels are significantly elevated in the cerebral cortex and/or hippocampus of AD model mice, possibly due to the elevated expression of the m6A methyltransferase and/or the decreased expression of the m6A demethylase, which might be associated with the membrane synaptic growth [31,34]. It has been reported that the methyltransferase-like 3 (METTL3) may be downregulated at the hippocampus in human AD samples, and upregulation of METTL3 could promote autophagic p-Tau clearance and ameliorate AD both in vitro and in vivo [35]. In addition, the m6A could control protein levels of key genes involved in AD-associated pathways [33,36].

### 2.2. Parkinson’s Disease

Parkinson’s disease (PD) might be the second most-frequent neurodegenerative disease after AD. It has been shown that multiple m6A methylated proteins are associated with PD. The m6A modifications could also regulate dopaminergic signaling. For example, the deletion of METTL14 in the substantia nigra could reduce the level of m6A mRNAs, and might impair the autonomic activity in mice [37]. Overexpression of the fat mass and obesity-associated (FTO) gene and m6A inhibitor cycloleucine could reduce m6A levels in PD models [38]. It has been shown that manganese exposure is a major environmental cause of PD. Remarkably, FTO overexpression could also diminish the manganese-induced cytotoxicity, and thereby could improve the symptoms of PD [39]. Accordingly, it is suggested that some inhibitors of FTO might be utilized for the treatment of PD. For example, entacapone is a catechol-O-methyltransferase inhibitor approved as an adjunctive therapy in combination with levodopa for PD treatment which could promote the modification of the target gene forkhead box protein O1 (FOXO1)-m6A by decreasing FTO expression [40].

### 2.3. Neurodegenerative Disorders and Aging

Huntington’s disease is an autosomal dominant neurodegenerative disorder, which is characterized by choreiform movements, cognitive deficits, and/or psychiatric symptoms [41]. These impairments may be attributed to hippocampal dysfunction and/or corticostriatal dysfunction [42]. Altered m6A RNA methylation as a unique hallmark of Huntington’s disease might contribute to hippocampal memory deficits in model mice of Huntington’s disease [43]. Huntington’s disease is an autoimmune disease of the nervous system mainly caused by the autoantibodies against the acetylcholine receptor (AChR) in the postsynaptic membranes of the neuromuscular junction [44]. It has been suggested that myasthenia gravis may be a complex multifactorial disease involving environmental, genetic, and/or immunological factors. Among them, the m6A regulators might play imperative roles in the pathogenesis of myasthenia gravis [45]. Multiple sclerosis is a neuro-inflammatory disease that affects the brain, spinal cord, and optic nerves [46]. Unfortunately, there is also no permanent cure for this neurological disorder. Optimal treatment regimens should be agreed upon for a good quality of life (QOL) [47]. The m6A RNA modifications might be closely associated with the development of multiple sclerosis [48]. It is noteworthy that cellular senescence is an important component of the aging process. It has been suggested that aging could definitely affect m6A RNAs methylation in the hippocampal regions [49]. In addition, there are aging-related differences in the modification level of the epigenome m6A [50]. Additionally, the reversibility of the m6A modification in aging may indicate its possibility for delaying aging.

## 3. ROS, Inflammation and m6A mRNAs

**Figure 2:**
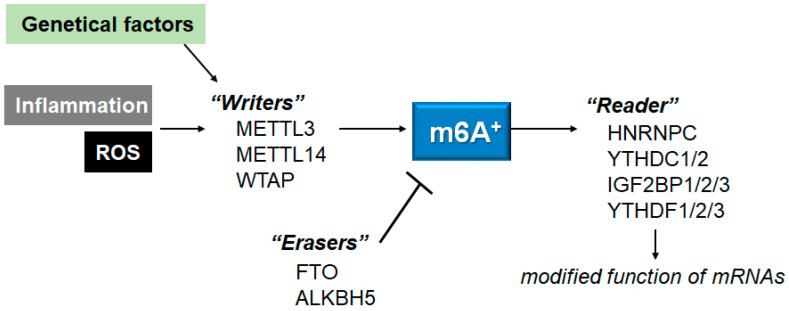
Schematic representation of the players involved in the m6A RNAs methylation. The m6A modification is regulated by methyltransferases “writers” and demethylases “erasers”. The m6A-RNAs binding proteins are called “readers”. Example molecules are also shown for each player. Genetic factors and/or inflammation with ROS may affect the function of these players. The arrowhead means stimulation, and the hammerhead represents inhibition. Note that some critical pathways have been omitted, for clarity.

The m6A modification is the most dynamic and reversible epigenetic modification of eukaryotic mRNAs [51], which could be regulated by methyltransferases and/or demethylases [52]. Methyltransferases, also called “writers”, may be composed of METTL3, methyltransferase-like 14 (METTL14), and/or Wilms tumor 1-associated protein (WTAP), which could catalyze the methylation of N6-adenosine with the function of methyl connection [53]. The METTL3 might be the catalytic subunit, while the METTL14 may be involved in the stability of the complex, as well as the RNA recruitment. The WTAP may also be required for the recruitment of mRNA. Among them, the METTL3 has been described as playing imperative roles in many pathological processes, predominantly in inflammatory and/or autoimmune responses [53]. m6A-RNAs binding proteins, also called “readers”, may generally contain heterogeneous nuclear ribonucleoprotein C (HNRNPC), YTH domain-containing protein 1/2 (YTHDC1/2), insulin-like growth factor 2 mRNA-binding protein 1/2/3 (IGF2BP1/2/3), and/or YTH domain-containing family protein 1/2/3 (YTHDF1/2/3). Reader molecules might be prepared for distinguishing the m6A motif, thereby accomplishing the modification of function on the m6A-RNA [54]. Additionally, the m6A modification could be detached by demethylases, also known as “erasers”, including alkB homolog 5 (ALKBH5) and/or FTO (Figure 2). For example, the FTO could favorably demethylate the m6A located nearer to the mRNA cap.

For example, the half-life of myosin heavy chain 3 (Myh3) mRNA could be significantly decreased after METTL3 deletion, in which Myh3 might have several potential m6A modification sites. Downregulation of METTL3 could reverse the lipopolysaccharide (LPS)-induced myocardial cell damage, largely by increasing Myh3 mRNA stability [55]. In addition, the deletion of METTL3 could also increase the expression of myeloid differentiation factor 88 (MyD88), which may prevent the activation of NF-κB signaling in the LPS-treated cells [56]. Similarly, the silencing of FTO could suppress the proliferation and/or invasion of cervical cancer cells via the m6A modification of the myelocytomatosis oncogene (Myc) and the zinc finger E-box-binding homeobox 1 (ZEB1) [57]. The silencing of FTO could also inhibit the NLRP3-mediated IL-1β expression through the modification of the NF-κB signaling [58], which may be related to the protection of cells.

Methylation put in by the “writers” could be reversed by “erasers” [59], which might be controlled for healthy cellular homeostasis. Therefore, dysregulations of m6A might be linked to the perturbations of cell death and/or proliferation in diverse diseases [60]. Accordingly, m6A modifications are potential novel diagnostic and/or therapeutic targets for several diseases, including neurodegenerative disease [61]. As mentioned above, the m6A modification may be vigorously controlled by RNA methyltransferases (also known as “writers”), RNA demethylases (“erasers”) and RNA-binding proteins (“readers”) [62]. Interestingly, the m6A level of RNAs may be significantly increased with the treatment of LPS [63]. In addition, METTL3 expression might also be upregulated via the increased ROS production [64]. For example, the ROS content might be elevated with the concomitant increase in m6A methylation in the liver after LPS treatment [65]. Therefore, the m6A methylation of RNAs could be modulated under oxidative stresses [65]. Furthermore, ROS could significantly increase the expression of YTHDF2, with the associated elevation of m6A methylation. Gathering this evidence has suggested that the alteration of the m6A modification may be a prevalent phenomenon under oxidative stress conditions. Therefore, immunological and/or inflammatory stresses could affect the m6A level of mRNAs [66]. In general, ROS could regulate major epigenetic processes in various cells [67,68]. In particular, the change in ROS levels might contribute to the m6A RNA methylation [69]. Consequently, the m6A methylation may be related to the increased levels of ROS content.

## 4. m6A RNA Modifications in Autophagy

Epigenetic modifications such as histone modifications, DNA methylations, and/or RNA methylations could totally control the expression of several genes involved in autophagy [70]. For example, it is noteworthy that the alteration of unc-51-like autophagy-activating kinase 1 (ULK1) mRNA expression could be post-transcriptionally altered by the m6A RNA modification, triggering a considerable inhibition of autophagy [71]. Subsequently, many investigations have revealed the effects of m6A modification in the autophagy-related mechanisms [20,72]. The m6A modification could convey inhibitory effects on autophagy, in which the METTL3 might be the key factor involved in the aberrant modification of m6A [21,73]. Sometimes it could promote autophagy instigation [23,74]. In these ways, the m6A modification might play a crucial role in regulating autophagy, which may be dependent on the disease condition. Therefore, comprehension of the m6A methylation machinery in autophagy regulation may be essential for development of therapeutic strategies [22,75].

The YTHDF2 could catch eIF4G1 transcripts with m6A methylation, and may induce the mRNA degradation, thereby promoting autophagy [76]. In addition, the m6A modification could also enhance the stability of zinc finger NFX1-type containing 1 (ZNFX1) antisense RNA 1 (ZFAS1) [77]. In more detail, for neural cells, the lnc RNA of ZFAS1 is upregulated in neural progenitor cells [77], which could regulate the expression of ATG10, and control the autophagy by preventing the PI3K/AKT pathway from stimulating the migration and/or proliferation of neural progenitor cells [77]. Similarly, the YTHDF1 could enhance the translation of ATG2A and ATG14 autophagy-related genes by binding to the m6A-modified mRNA of ATG2A and ATG14, consequently assisting autophagy [78]. The YTHDF1 could also support the translation of the other m6A-modified mRNAs [78]. Therefore, the YTHDF1-knockout may weaken the ability of RNA-binding proteins to recognize m6A, thus impeding mRNA translation, and interrupting downstream molecular functions. In general, autophagy preserves cellular homeostasis, and might support cells to react to various stresses by recycling the impaired organelles, proteins, lipids, and other cellular components. Neuron-protective autophagy could promote the survival of neurons [79]. Now, a huge number of investigations have confirmed that m6A modification could control the initiation and/or activation of autophagy by modifying the expression of ULK1, ATG5, and/or FIP200 [80]. Accordingly, m6A adjustments could control the expression of several genes related to autophagy. Elevated m6A modifications could also stimulate the formation of autophagosome, as well as the lysosomal function [81,82]. Therefore, m6A modification and impaired autophagy might be related to the progress of neurodegenerative diseases, in which the m6A-autophagy partnership could play crucial roles.

By enhancing autophagy, cellular senescence and/or differentiation could be meaningfully refreshed [83]. Atypical autophagy may bring about a range of diseases containing neuron degenerative disorders, cerebral ischemic injury, innate immunity diseases, myocardial dysfunction, cardiomyopathy, nonalcoholic fatty liver disease, hypertensive nephropathy, cancers, and various inflammatory diseases [84]. Suppression or up-regulation of autophagy by m6A modification might depend on the level of m6A methylation and/or the role of downstream targets. Interestingly, defective autophagy may be related to the growth of local gram-negative bacteria in the intestine [85]. Although this research has been intensely dedicated to the molecular mechanisms involved, further detailed investigations are immediately required to elucidate the exact interaction between m6A modification and autophagy under diverse pathological conditions. The relation between m6A methylation and autophagy might be a remarkable topic in cellular and molecular biology research.

## 5. Possible Therapeutics for Neurodegenerative Diseases

Several patterns of neurodegeneration may be restricted from altered pathological mechanisms. For example, AD is determined by the increase in pathologic amyloid, and eventually directs to dementia, whereas PD is determined by the loss of dopaminergic neurons, and primarily directs to several extrapyramidal symptoms. As aberrant autophagy has been observed in neurodegenerative diseases, including AD, PD, HD, and/or amyotrophic lateral sclerosis (ALS) [86], something to modify the autophagy might contribute to the development of treatment for these neurodegenerative diseases. In this regard, as gut microbiota can play an imperative role in the response of inflammation, autophagy, oxidative stress, and/or cellular apoptosis, even for organs distant from the gut such as the kidney and/or brain [87,88], the gut microbiota might contribute to improve the pathology of neurodegenerative diseases. The comprehensive understanding of the autophagy regulation might help in interpreting their impact on human diseases and may aid in devising future therapeutic strategies.

In general, neurodegenerative diseases are mostly hard to block, and only symptomatic treatments are available. Accordingly, finding an effective treatment is very important. The recognition of m6A mRNA methylation has become a new feature in post-transcriptional gene expression machineries [59]. Several animal experiments have also proposed the alteration roles of m6A methylation on various neuronal function and/or neural patterning [9,89]. However, the significance of m6A RNAs methylation for cognitive dysfunction might remain mainly unknown. As certain m6A-related regulators might serve as novel therapeutic strategies for neurodegenerative diseases, the organized assessment of m6A modifications could set an important foundation for comprehending the physical characteristics of neurodegenerative diseases, including cognition. The evaluation might contribute to guiding even more effective therapeutic strategies for dementia. Investigations of the link between m6A methylation and aging may also provide novel therapeutic targets with important medical implications against aging. In particular, the comprehension of interplay between m6A modifications and autophagy may assist in developing future therapeutic strategies for definitive anti-aging. In line with this, the m6A modification could alleviate the premature senescence of stem cells via the novel epitranscriptional mechanism [90]. In addition, manipulation of the autophagy pathway may also be a promising tactic to prevent the progress of aging and/or aging related diseases. In fact, it has been shown that small non-coding RNAs (18–24 nucleotides) such as microRNAs (miRNAs) could meaningfully regulate autophagy in relation to age-related diseases [91]. miRNAs could also affect the expression of multiple genes, which might play diverse important roles in anti-aging. The miRNAs can bind to the 3′UTR of several target genes and promote the degradation of objective genes, thus regulating the translation of certain mRNA. It would be stimulating to examine the effects of these miRNAs on autophagy in the development of neurodegenerative diseases. For example, the deregulation of definite miRNA could produce a distinctive character for neurodegenerative disorders, which could probably lead to a basic diagnosis. Adjusting conventional symptomatic treatments has become considerably effective for conservative therapy in many cases; however, disease-modifying therapies are still required. Few have identified inhibitors absolutely targeting m6A regulatory molecules, while gene treatments with genome-editing technology for hereditary disorders have attracted attention.

Interestingly, it has been shown that the modulation of m6A levels could become a novel therapeutic target for dealing with the SARS-CoV-2 infection [92]. Previous studies have found that the natural product rhein competitively binds to the FTO active site in vitro [93], which could improve virus-induced lung injury [94]. In this way, the m6A modification could play a key role in the incidence and/or development of several human diseases. However, the mechanisms are not completely clear. In addition, the exact function of each m6A factor might be different during the stages of disease. Therefore, it might be a challenge to apply the m6A technique in various disease therapies, including neurodegenerative diseases. Interestingly, it has been suggested that the persisting commensals could be involved in the gut microbiota-mediated regulation of m6A modification [95].

## 6. Future Perspectives Involved in the Therapeutics

**Figure 3:**
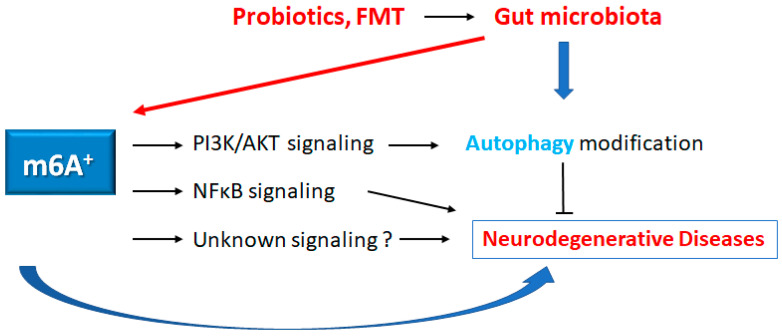
Schematic representation of the possible inhibitory tactics against the pathogenesis of neurodegenerative diseases. Some kinds of probiotics and/or fecal microbiota transplantation (FMT) could contribute to the alteration of the gut microbial community for the alteration of autophagy and/or m6A RNAs methylation, which might be beneficial for the treatment of neurodegenerative diseases. The arrowhead indicates stimulation, whereas the hammerhead shows inhibition. Note that several important activities such as cytokine-induction, ROS production, and/or inflammatory reactions have been omitted, for clarity.

This research topic is directed to exploring the therapeutic effect and mechanism action as an integrative medicine for the improvement in patient healthcare of neurodegenerative diseases. The m6A, as the most common posttranscriptional modification of eukaryotic mRNAs, has been shown as playing significant roles in the gut and/or the brain [95]. Interestingly, *A. muciniphila* and *L. plantarum* in gut microbiota could affect specific m6A modifications in mice, which may emphasize epitranscriptomic modifications by commensal bacteria [95]. Studies have shown that epigenetics of m6A mRNAs could play a part in the regulation of incidence and/or the development of a variety of diseases, including neurodegenerative diseases, in which gut microbiota and RNA epigenetics could construct a complicated cross regulatory network [29,96]. Interestingly, the removal of YTHDF1 could stimulate *A. muciniphila* colonization to increase anti-inflammatory effects as a feedback system, by promoting the expression of Foxp3 via the m6A modification [97]. Using this concept, it is possible to treat neurodegenerative diseases and/or neurodegenerative disorders with probiotics and/or fecal microbiota transplantation (FMT) [98,99] (Figure 3). For example, the FMT has a mitigating effect on high-fat diet-induced obesity, which may be due to the striking effects of FMT on the microbial composition. Interestingly, the mitigating effect with FMT could also alter intestinal lipid metabolism, in addition to the alteration of m6A methylation levels, to accomplish the opposition to obesity [100]. Again, it has been shown that the gut microbiota could be involved in the regulation of m6A modification [95,100] (Figure 3). Furthermore, we have already suggested that “engram theory”, with the concept of improved gut microbiota, might become a promising tactic for the treatment of neurodegenerative disorders [99,101]. Accordingly, non-invasive brain stimulation, with the alteration of gut microbiota, appears to possess much potential. Intrinsically, emphasizing the latest research results and/or assembling the available evidence has been more imperative than ever. However, more in-depth explorations are required to develop novel therapeutic strategies based on the interaction among m6A methylation, gut microbiota and/or autophagy, in order to support further understanding and ultimately provide new methods for the prevention and/or treatment of neurodegenerative diseases. It would also be mandatory to explore the precise molecular mechanisms of these “theories” for the development of innovative treatments.

## Data Availability

Data sharing not applicable.

## Abbreviations

AChR: acetylcholine receptor; AD: Alzheimer’s disease; ALS: amyotrophic lateral sclerosis; CNS: central nervous system; circRNA: circular RNA; FMT: fecal microbiota transplantation; FTO: fat mass and obesity-associated; HD: Huntington’s disease; lncRNAs: long non-coding RNAs; m6A: N6-methyladenosine; METTL3: methyltransferase-like 3; METTL14: methyltransferase-like 14; miRNA: microRNA; mRNA: messenger RNA; Myc: myelocytomatosis oncogene; ncRNA: non-coding RNA; MG: myasthenia gravis; MS: multiple sclerosis; ncRNA: non-coding RNA; PD: Parkinson’s disease; QOL: quality of life; UTR: untranslated region; WTAP: Wilms tumor 1-associated protein; ZEB1: Zinc finger E-box-binding homeobox 1.
